# Bone sporotrichosis: 41 cases from a reference hospital in Rio de Janeiro, Brazil

**DOI:** 10.1371/journal.pntd.0009250

**Published:** 2021-03-17

**Authors:** Vanessa Ramos, Guis S-M. Astacio, Antonio C. F. do Valle, Priscila M. de Macedo, Marcelo R. Lyra, Rodrigo Almeida-Paes, Manoel M. E. Oliveira, Rosely M. Zancopé-Oliveira, Luciana G. P. Brandão, Marcel S. B. Quintana, Maria Clara Gutierrez-Galhardo, Dayvison F. S. Freitas

**Affiliations:** 1 Pós-Graduação em Medicina Tropical, Instituto Oswaldo Cruz, Fundação Oswaldo Cruz, Rio de Janeiro, Brazil; 2 Instituto Nacional de Infectologia Evandro Chagas, Fundação Oswaldo Cruz, Rio de Janeiro, Brazil; 3 Instituto Oswaldo Cruz, Fundação Oswaldo Cruz, Rio de Janeiro, Brazil; Yale University School of Medicine, UNITED STATES

## Abstract

**Background:**

Bone sporotrichosis is rare. The metropolitan region of Rio de Janeiro is hyperendemic for zoonotic sporotrichosis and the bone presentations are increasing.

**Methods:**

We studied a retrospective cohort of 41 cases of bone sporotrichosis, diagnosed from 1999–2016. The inclusion criteria was fungal culture isolation from any clinical specimen associated to bone involvement (radiography and/or computed tomography) compatible with fungal osteomyelitis or histopathological findings of bone material compatible with sporotrichosis. Molecular identification was performed when possible.

**Results:**

Male patients represented 58.5% of the cases, with a cohort median age of 43 years. Immunosuppressive conditions were present in 68.3% of the patients, mostly HIV coinfection (51.2%). Multifocal bone involvement (more than one anatomical segment) was diagnosed in 61% of the patients, while 39% presented unifocal involvement. The bones of the hands were the most affected (58.5%), followed by the feet (41.5%) and tibia (26.8%). Multifocal group was characterized by a higher proportion of males (p = 0.0045) with immunosuppressive conditions (p = 0.0014). Amphotericin B followed by oral itraconazole was the main treatment, with a median time of 16.7 months (1.5 to 99.2 months), and cure of 53.7% of the patients (84.6% of immunocompetent and 39.3% of immunocompromised patients). Sequelae occurred in 12.2% of the patients—amputations (7.3%) and ankylosis (4.9%), while 22% died in the course of the disease. *Sporothrix brasiliensis* was the causative agent in all the 9 (22%) performed cases.

**Conclusions:**

Bone sporotrichosis is a chronic, challenging condition with prolonged treatment, often with poor results and sequelae.

## Author summary

Sporotrichosis is a subcutaneous mycosis, more common in tropical and subtropical countries, and the involvement of bones is rare. The metropolitan region of Rio de Janeiro is hyperendemic for zoonotic sporotrichosis and the bone presentations are increasing. We evaluated 41 cases of bone sporotrichosis at a reference center in infectious diseases. There was a predominance of men with immunosuppressive conditions, notably HIV infection and alcoholism. Immunosuppression was related to multifocal bone involvement and more severe disease. This form of the disease is a chronic, challenging condition with prolonged treatment, often with poor results and sequelae.

## Introduction

Sporotrichosis is a worldwide subcutaneous mycosis, especially in tropical and subtropical regions [1]. The causative fungi, *Sporothrix* spp., live associated with plants, soil or decomposing organic material. The classic transmission is through traumatic inoculation of the fungus in the skin [2] and the metropolitan region of Rio de Janeiro is hyperendemic for cat-transmitted zoonotic sporotrichosis caused, in most cases, by *Sporothrix brasiliensis* [3–4]. The cutaneous forms usually account for more than 90% of the cases [2,3,5]. Osteoarticular involvement is the most common extracutaneous manifestation of sporotrichosis [2], nevertheless, it is considered rare [6–14].

Bone sporotrichosis occurs by contiguity or hematogenous spread, presents with an indolent course of pain and limitation of articular movement [15], alone or as part of a disseminated infection [6]. Its treatment is longer and requires a higher daily dose of antifungals, compared to cutaneous sporotrichosis. Itraconazole 400 mg/day is recommended for at least 12 months and, in more severe cases, the use of intravenous amphotericin B (AMB) may be necessary [16].

We evaluated the socio-demographic and epidemiological characteristics, and the clinical evolution of the patients with bone sporotrichosis, in a reference hospital, aiming to describe the cases and to find explanatory variables.

## Methods

### Ethics statement

All procedures performed were in accordance with the ethical standards laid down in the 1964 Declaration of Helsinki and its later amendments, as well as the Brazilian ethical standards—Resolution (CNS 466/12). The Instituto Nacional de Infectologia Evandro Chagas (INI) Review Board approved the study under the number 64068717.4.0000.5262. Written consent was waived, justified by the difficulty in obtaining it from most of the patients, and based on the compromise and responsibility of the principal investigator in anonymizing and protecting the patients’ personal data.

### Place of study, patients, and study design

INI, Fundação Oswaldo Cruz (Fiocruz), Rio de Janeiro, Brazil, is a reference center for the treatment of sporotrichosis in the state of Rio de Janeiro and, since the beginning of the increase in the number of cases of human sporotrichosis, in the late 90s, patients are followed up in a cohort. From this primary cohort, we selected patients with culture-proven sporotrichosis from any clinical specimen and associated bone involvement, from 1999 to 2016. Patients with other culture-proven causes of osteomyelitis were excluded from the study.

### Patient management

Patients were submitted to clinical evaluation, mycological examination (direct microscopy and culture) of clinical specimens and blood tests (blood count, biochemistry, liver function, erythrocyte sedimentation rate (ESR), and high sensitivity C-reactive protein (hs-CRP)).

Investigation of bone lesions was performed in patients with exuberant cutaneous lesions adjacent to bone surfaces (usually associated with pain, edema, and limitation of movement) and in those with disseminated cutaneous lesions or disseminated disease. In the first indication, local bone radiography was done and, in the second, a bone screening (total skeletal radiographs or bone scintigraphy) was performed to search for asymptomatic lesions. Regarding treatment, at the INI, patients with cutaneous forms receive oral itraconazole, whereas patients with disseminated sporotrichosis receive AMB up to clinical improvement, complemented by oral itraconazole, until clinical cure [16].

### Molecular identification

Nine of the patients’ *Sporothrix* clinical isolates could be recovered at the institutional Laboratory of Mycology and the species was identified by the T3B PCR fingerprinting, as previously described [17].

### Definition of bone sporotrichosis

Bone involvement was defined by imaging (primarily radiography and computed tomography) compatible with fungal osteomyelitis, analyzed by two independent radiologists and/or histopathological findings of bone material compatible with sporotrichosis. The healing of bone lesions was determined by normalization of the bone images or estimated by stabilization of the lesions through comparative images every three-six months.

### Data collection and statistical analysis

We reviewed the medical charts of the selected patients for the collection of socio-demographic, epidemiological, clinical and laboratory data. The data were entered into a database in the program FormSUS (service for the creation of forms of public access of the Brazilian National Health System) and analyzed with the assistance of the library of the R program, version 3.3.0. Contingency tables and association tests (qui-square and Fisher exact) were used to compare groups.

Univariate and multivariate analyses were performed using the Cox model of proportional risks, considering the time the patient was under treatment until the time of cure, and skin color, sex, immunosuppression, form of bone involvement, cat bite and alcoholism as predictors. For those patients who did not cure, such as those who died, those who lost follow-up or those still in treatment, the curve was censored at such moments (date of death, date of loss of follow-up and date of analysis).

## Results

**Graph 1:**
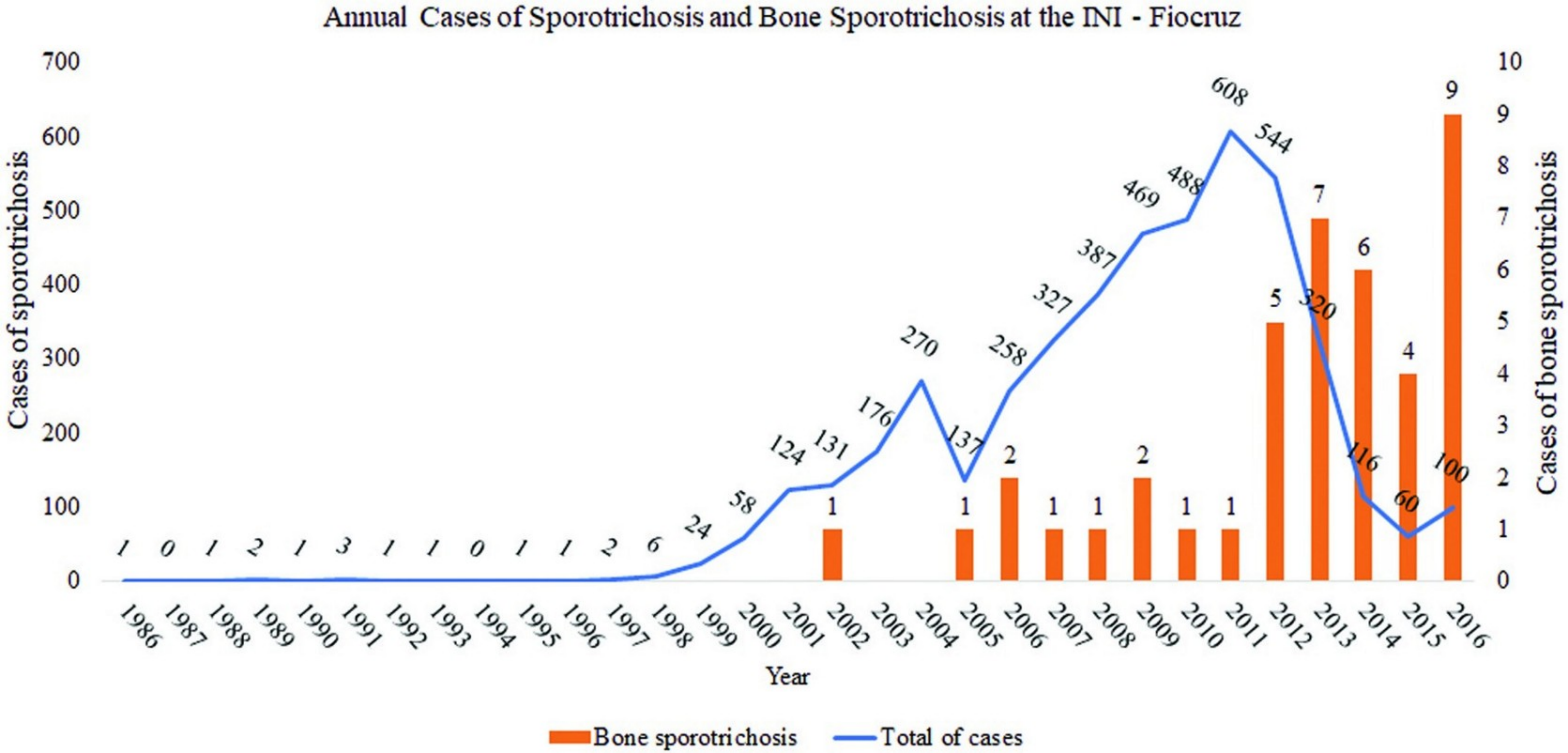
Number of cases of sporotrichosis and bone sporotrichosis seen historically at the INI-Fiocruz (1986 to 2016). (Source: Electronic data system of patients and database of the Laboratory of Clinical Research in Infectious Dermatology of the INI).

Forty-one cases of bone sporotrichosis were included and represented 0.9% of the 4,617 cases of sporotrichosis treated at the INI-Fiocruz during the studied period, mostly concentrated in the last decade (Graph 1).

### Socio-demographic and epidemiological characteristics

Male patients represented 58.5% (n = 24) of the cases, while 41.5% (n = 17) were female. Non-white patients were 73.2% (n = 30, 23 brown and 7 black) and white patients, 26.8% (n = 11). The median age was 43 years (range: 16–79 years). Regarding the schooling: 58.5% (n = 24) studied up to 9 school years and 34.1% (n = 14) had more than 9 school years, including 7.3% (n = 3) with a bachelor’s degree. In 7.3% (n = 3), the schooling was unknown. Most of the patients came from the city of Rio de Janeiro (n = 17, 41.5%), mainly from the north and west zones of the city, followed by municipalities in the capital’s metropolitan region (Duque de Caxias: 17.1%, n = 7 and Belford Roxo: 9.8%, n = 4). The most prevalent occupations (48.8%, n = 20) were those related to household activities, such as housewife, housemaid, unemployed, retired and student. Contact with domestic cats was reported by 70.7% (n = 29) of the patients and, within this group, bites and/or scratches were reported in 41.4% (n = 12).

### Clinical characteristics

Table 1 presents clinical data of the patients. In brief, the initial clinical presentation of sporotrichosis was the disseminated cutaneous form in 70.7% of the cases (n = 29), followed by lymphocutaneous (22%, n = 9) and fixed cutaneous (3%, n = 3). Immunosuppressive conditions, present in 68.3% (n = 28) of the patients, included human immunodeficiency virus (HIV) infection (51.2%, n = 21), alcoholism (22.0%, n = 9), a combination of both (23.8%, n = 5), malnutrition, and chronic use of corticosteroids (4.9%, n = 2, each). Diabetes mellitus, although a potential immunosuppressive condition, was not considered so in this study since patients were stable for this comorbidity.

**Table 1:** Description of the initial clinical form, comorbidities and bones affected, of the patients with bone sporotrichosis, treated at the INI-Fiocruz, from 1999 to 2016.

**Case**	**Initial clinical form**	**Comorbidities**	**Affected bones**
**1**	DC / Nasal mucosa	HIV	Feet, R acromion, L ulna
**2 ^a^**	DC / Oral and nasal mucosa	HIV / Alcoholism	R hand (middle phalanx of 3^rd^ finger), R wrist, R ulna, R ankle, Knees
**3**	LC (R arm) / Choroiditis	HIV	L wrist, R radius, R ulna, R humerus, R foot, R ankle
**4 ^a^**	DC / Nasal and oral mucosa / Choroiditis	HIV	Feet, L clavicle, R radius, L wrist
**5 ^a^**	DC / CNS	HIV	Hands, R ankle, L clavicle, Feet, R wrist
**6 ^a^**	DC / Nasal and oral mucosa / Choroiditis / CNS	HIV	R hand (middle phalanx of 2^nd^, 3^rd^, 4^th^ and 5^th^ finger), R wrist, Tibias, Fibulas
**7 ^a^**	DC / Nasal mucosa	HIV	Wrists, Elbows, Knees, Ankles
**8**	LC (L hand)	HBP / DM	L hand (4^th^ Finger)
**9**	LC (L hand)	HBP	Distal phalanx of 5^th^ L finger
**10**	LC (R hand)	-	Distal phalanx of 2^nd^ R finger
**11**	DC	Alcoholism / Corticosteroids use	L olecranon
**12**	FC (R hand)	HCV	Distal phalanx of 1^st^ R finger
**13**	LC (R hand) / synovitis	HBP / DM	R wrist
**14**	LC (L hand)	-	Distal phalanx of 1^st^ L finger
**15**	DC / Nasal mucosa	HBP / DM	Proximal phalanx of 4^th^ R finger
**16**	DC	HBP	L metacarpi and phalanges, Middle phalanx of 4^th^ R finger
**17**	DC	-	R clavicle
**18 ^a^**	DC	Malnutrition	5^th^ L metatarsus, L tibia, L fibula
**19**	DC	HIV / Alcoholism	Feet (tarsi, L and R metatarsi, L calcaneus)
**20**	DC / Synovitis	Alcoholism	Tibias, R wrist, Hands (several phalanges), Feet
**21**	LC (R arm) / CNS	HIV	R hand (proximal phalanx of 2^nd^ finger)
**22**	LC (R arm)	HIV	Tibias and R calcaneus
**23**	DC	HIV	Hands (2^nd^ R finger and 5^th^ R metacarpus)
**24**	DC / Nasal mucosa	HBP / Malnutrition	Hands (3^rd^ R metacarpus and 2^nd^ L metacarpus)
**25**	DC / Nasal and oral mucosa / Synovitis	HIV	L foot (3^rd^ toe), R knee
**26 ^a^**	DC	HBP / DM	L wrist, Knees
**27**	DC	Alcoholism	L tibia and calcaneus
**28 ^a^**	DC / Nasal and oral mucosa	HIV / HBP	L hand (4th finger), L ankle, Knees
**29**	DC / Nasal and oral mucosa	DM / Lepra / Corticosteroids use	Hands (proximal and middle phalanges of 2^nd^ to 5^th^ R fingers and several phalanges of 2^nd^ to 5^th^ L fingers), R ulna, Feet, Tibias (distal extremities), Ischia, L femur
**30**	DC / Nasal and oral mucosa	HIV / Alcoholism	Hands (proximal phalanx of 5^th^ R finger, middle phalanx of 4^th^ R and L fingers, proximal phalanx of 3^rd^ L finger), Feet (5^th^ R metatarsus, L cuboid), Tibias
**31**	DC / Oral mucosa / Retinitis	-	2^nd^ R finger, Feet, Skull, Costal arches, Clavicles, R ulna, L radius, L fibula and tibia
**32**	DC	Alcoholism	R foot (distal phalanx of 2^nd^ toe)
**33**	LC (R hand and arm)	_	Distal phalanx of 2^nd^ R finger
**34**	DC / Nasal mucosa	HIV	Foot (1^st^ R metatarsus)
**35^a^**	DC	HIV / Alcoholism	L hand (proximal phalanx of 2^nd^ finger)
**36**	DC	HIV / Alcoholism	Hands (3^rd^ R metacarpus, 2^nd^ L metacarpus, proximal phalanx of 4^th^ L finger), R foot (1^st^ metatarsus), R elbow
**37**	DC	HIV	Hands (proximal phalanx of 3^rd^ and 5^th^ L fingers), Feet (5^th^ R metatarsus, R and L calcaneus), R tibia, R and L ulnas, R and L humeri
**38**	DC / Nasal mucosa / CNS	HIV	Hands (proximal phalanx of 2^nd^ L finger), L radius and ulna
**39**	FC (L wrist)	HBP / DM	L wrist
**40**	FC (R hand) / Nasal and oral mucosa	HIV / HBP / DM	Hands (middle phalanx of 3^rd^ R finger, proximal phalanx of 4^th^ L finger), L ulna, Feet, Tibias
**41**	DC	HIV	R ulna and radius

DC: disseminated cutaneous; LC: lymphocutaneous; FC: fixed cutaneous; CNS: central nervous system; R: right; L: left; HIV: human immunodeficiency virus; HBP: high blood pressure; DM: diabetes mellitus; HCV: hepatitis C virus.

a: Nine cases were previously published, exploring the bone or other impairments of sporotrichosis: case 2: as case 3 in [18]; case 4: as case 1 in [18]; case 5: in [12,19] and as case 7 in [20]; case 6: as case 2 in [18]; case 7: in [21]; case 18: in [10]; case 26: as case 6 in [20] and in [22]; case 28: as case 1 in [23] and as case 12 in [24] and case 35: as case 2 in [23] and as case 14 in [24].

### Mycological results

**Fig 1:**
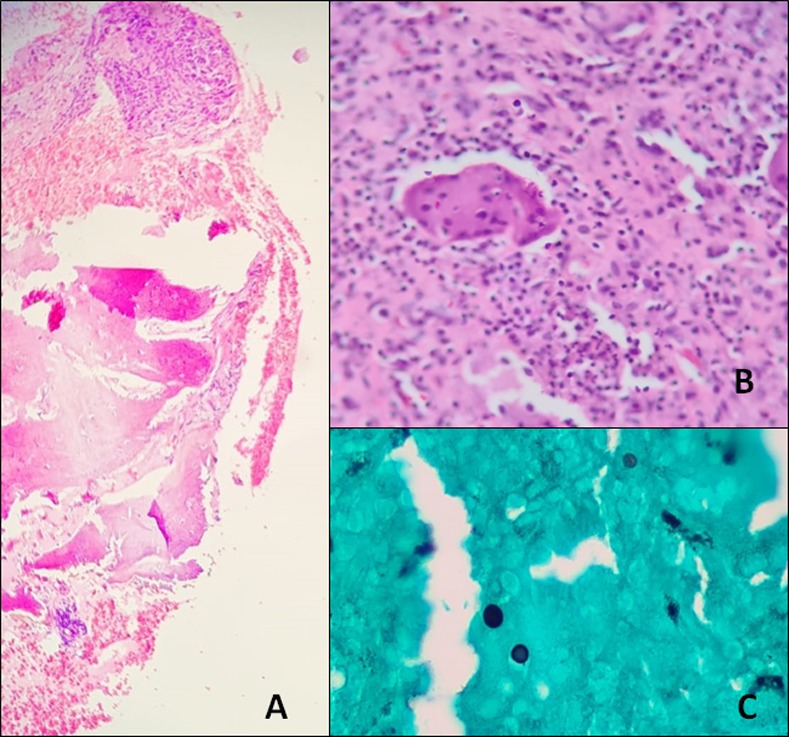
Bone sporotrichosis histopathology (case 31). A) Bone trabeculae next to connective tissue with chronic inflammatory process (Hematoxylin and eosin, 20X). B) Connective tissue with mononuclear inflammatory infiltrate and giant cell reaction (Hematoxylin and eosin, 40X). C) Yeast-like structures (dark rounded) (Grocott’s Methenamine Silver, 40X). (Source: courtesy of Dr. Janice Mery Chicarino de Oliveira Coelho).

For all patients, *Sporothrix* spp. was isolated from cutaneous specimens (lesion exudate or skin biopsy). Additionally, in 43.9% (n = 18) of the cases, the fungus was also isolated from other specimens: nasal swab (17.1%, n = 7), synovial fluid (12.2%, n = 5), oral swab (9.8%, n = 4), blood (7.3%, n = 3), bronchoalveolar lavage, lymph node, cerebrospinal fluid (4.9%, n = 2, each), and nasal biopsy, larynx, and urine (2.4%, n = 1, each). Bone biopsy was performed in 4 (9.8%) patients. One was the first case of our cohort (in 2002 –case 31), with a granulomatous inflammatory infiltrate and rare yeast-like structures in histopathology (Fig 1); the second (case 26) presented a chronic disease, with knee bone destruction and a biopsy was performed, with the isolation of the fungus in culture, despite an unspecific inflammatory process [22]; the third was already referred from another institution with the bone diagnosis (case 34), and the fourth (case 8) was submitted to a bone debridement and, due to the extensive destruction, the surgeon decided to amputate the fourth left finger with the visualization of the fungus in the histopathology.

### Molecular identification of the clinical isolates

The nine (22%) analyzed isolates were identified as *S*. *brasiliensis* by the T3B PCR fingerprinting. Four of these were previously reported (case 4: as case 1 in [18]; case 5: in [12,19] and as case 7 in [20]; case 6: as case 2 in [18] and case 26: as case 6 in [20] and in [22]). The other five patients, with the causative species still unpublished, are cases 8, 17, 28, 31 and 37.

### Bone involvement

**Fig 2:**
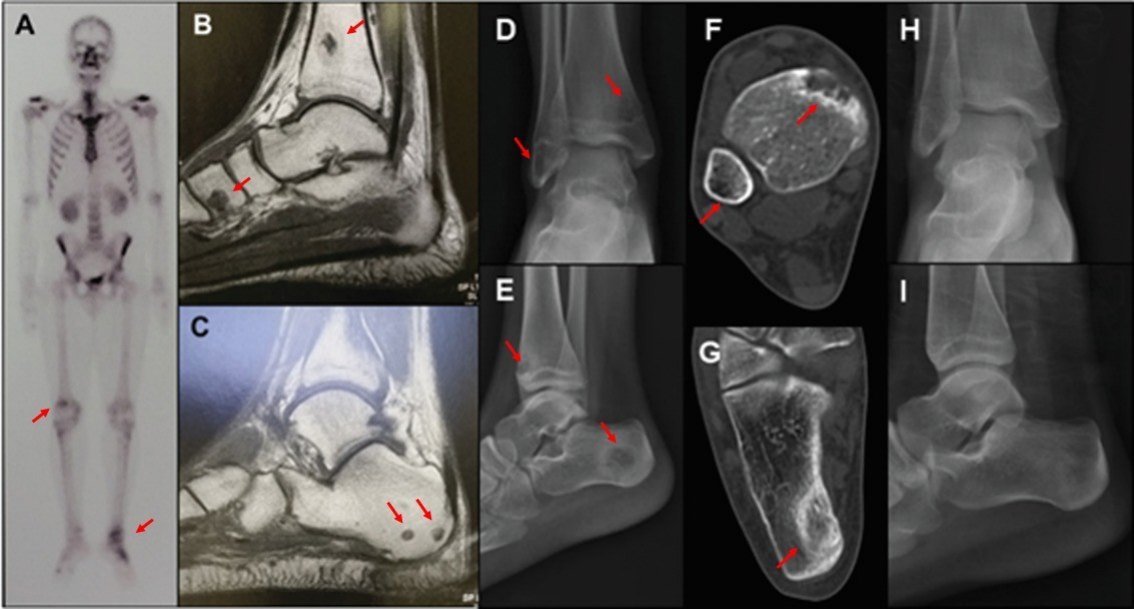
Different imaging diagnosis in bone sporotrichosis. A-C (case 40): A) Bone scintigraphy screening demonstrating uptake of the radiopharmaceutical in the right knee, left ankle, and foot. B-C) Magnetic resonance of the left ankle and foot—Round and well-defined lithic lesions in the tibia, calcaneus, and cuneiform. (Source: Laboratory of Clinical Research in Infectious Dermatology). D-I (case 22): D-E) Radiographs—Lytic lesions in the tibia, fibula, and calcaneus. F-G) Same lesions seen on computed tomography. H-I) Radiographs 11 months later, showing resolution of the lesions. The lesions are pointed by the red arrows. (Source: Service of Image of the INI).

In this series, the initial imaging diagnosis was by radiography (73.2%, n = 30), computed tomography (14.6%, n = 6), magnetic resonance (7.3%, n = 3) and bone scintigraphy (2.4%, n = 1) (Fig 2).

The most common radiological findings were the well circumscribed medullary lytic lesions with preservation of the cortical bone (80.5%, n = 33).

**Fig 3:**
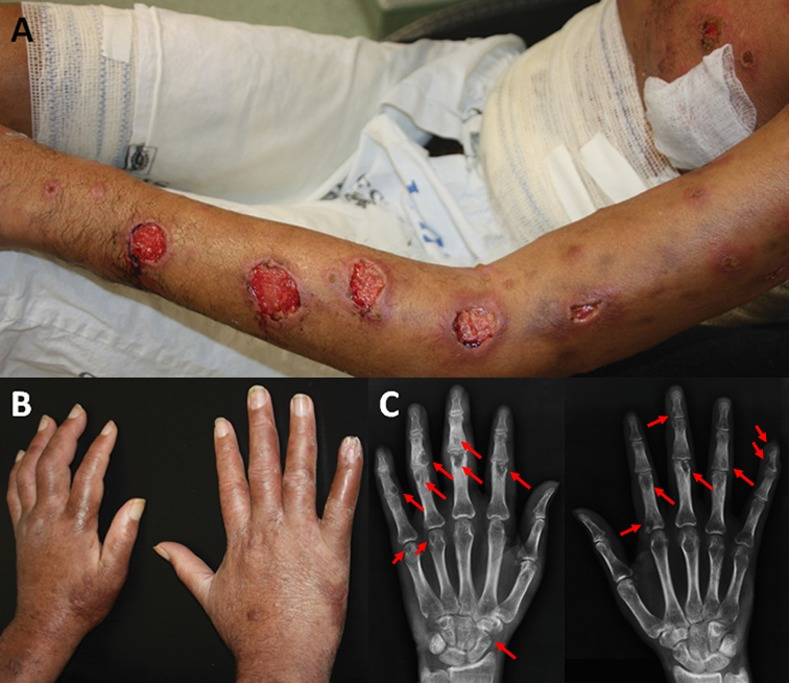
Multifocal form (case 29). A) Extensive ulcerated sporotrichosis lesions on the left upper limb and trunk. B) Hands with edema, more noticeable to the right (clinical images were inverted to correspond to the radiographs). C) Radiography—Multiple lytic lesions and bone erosions in both hands (arrows). (Source: A-B—Images by Dr. Marcelo Rosandiski Lyra; C–Service of Image of the INI).

**Fig 4:**
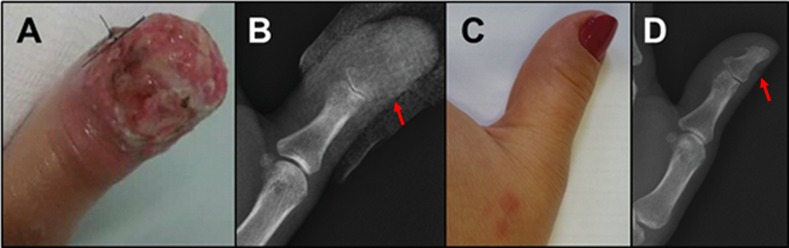
Unifocal form (case 12). A) Exuberant ulcerated lesion of fixed cutaneous sporotrichosis, with first right finger volume increase. B) Radiography—Destruction of the distal phalanx of the first right finger, with swelling of soft tissue (arrow). C-D) Clinical and radiological improvement after 9 months (arrow). (Source: A, C—Laboratory of Clinical Research in Infectious Dermatology; B, D—Service of Image of the INI).

Multifocal bone involvement (lesions in more than one anatomical segment) was diagnosed in 61% (n = 25) of the patients (Fig 3), while 39% (n = 16) presented unifocal involvement (lesions in only one anatomical segment, Fig 4).

The bones of the hands were the most affected ones (58.5%, n = 24), followed by the bones of the feet (43.9%, n = 18) and tibia (26.8%, n = 11, Table 1). The feet, in the multifocal involvement, were affected in 64% of the cases whereas in unifocal involvement, this occurred in 12.5%. So, the calculated risk ratio for a patient with the multifocal involvement to present lesion in the bones of the feet is 5.12 (95% CI: 1.36–19.35; p = 0.001), when compared to the group with unifocal involvement.

**Fig 5:**
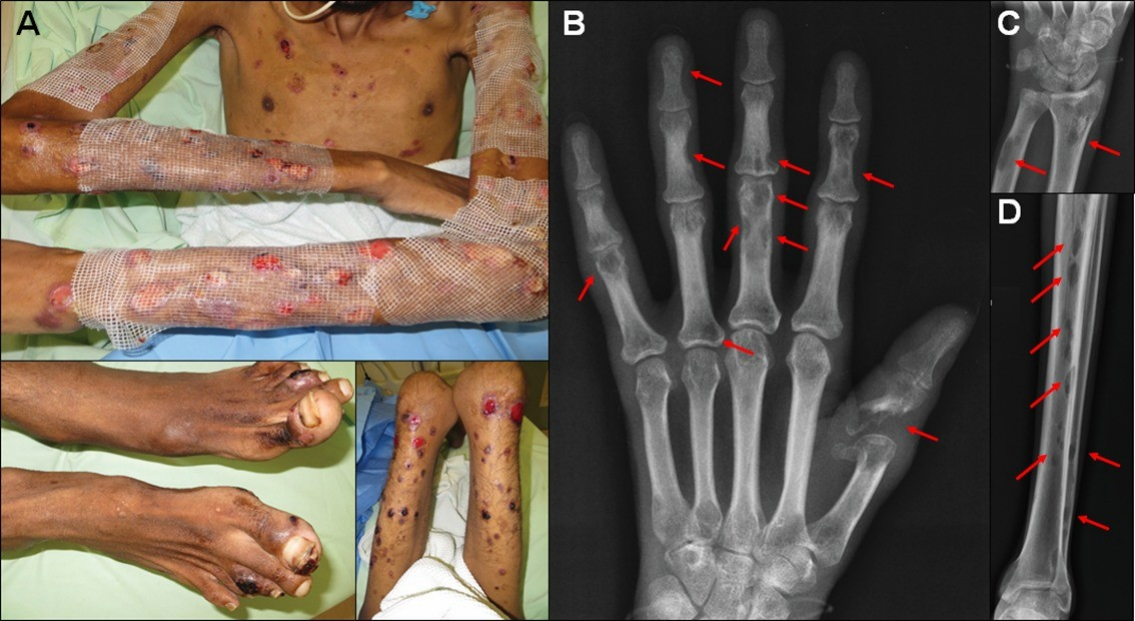
Patient infected with HIV, multifocal bone sporotrichosis (case 6). A) Patient with disseminated sporotrichosis and advanced AIDS. Multiple pleomorphic ulcerated lesions. Radiographs—B) Multiple lytic lesions with destruction of the proximal phalanx of the first right finger. C) Lytic lesions in the right wrist. D) Lytic lesions along the left tibia and fibula. The lesions are pointed by the red arrows. (Source: A—Laboratory of Clinical Research in Infectious Dermatology; B-D—Service of Image of the INI).

The comparative analysis by association tests of the multifocal and unifocal groups (Table 2) showed that the multifocal group was characterized by a higher proportion of males (p = 0.0045) with immunosuppressive conditions (p = 0.0014), notably HIV infection (p = 0.0109). When HIV-infected patients were compared to other patients, they were younger (median age: 38 years) and predominantly men (76.2%, n = 16). All these patients had disseminated cutaneous lesions, with multifocal bone involvement in 81% (n = 17, Fig 5). The diagnosis of HIV was concomitant with the diagnosis of sporotrichosis (or during the investigation of cutaneous sporotrichosis lesions) in 38.1% (n = 8) of the cases and 85.7% of the coinfected patients (n = 18) did not use antiretroviral therapy (they were either in treatment abandonment or had not started it yet). The CD4^+^ T cell count at the time of the diagnosis of sporotrichosis ranged from 1 to 348 cells/mm^3^, with 81% (17/21) bellow 200 cells/mm^3^, median of 46 cells/mm^3^ and viral load between undetectable and log 5.74.

**Table 2:** Association of selected variables with the type (unifocal/multifocal) of bone involvement, for patients with bone sporotrichosis treated at the INI-Fiocruz, from 1999 to 2016.

	Bone Involvement—N (%)		
	Unifocal	Multifocal	pv
	16	25	
**Immunosuppression**			**0.0014^a^**
**Yes**	6 (37.5)	22 (88.0)	
**No**	10 (62.5)	3 (12.0)	
**Sex**			**0.0045^b^**
**Male**	5 (31.2)	19 (76.0)	
**Female**	11 (68.8)	6 (24.0)	
**Skin color**			0.6092^b^
**Non-white**	11 (68.8)	19 (76.0)	
**White**	5 (31.2)	6 (24.0)	
**Cat bite**			1^a^
**Yes**	2 (12.5)	3 (12.0)	
**No**	14 (87.5)	22 (88.0)	
**HIV infection**			**0.0109^a^**
**Yes**	4 (25.0)	17 (68.0)	
**No**	12 (75.0)	8 (32.0)	
**Alcoholism**			1^a^
**Yes**	3 (18.7)	6 (24.0)	
**No**	13 (81.3)	19 (76.0)	
**Municipality**			0.6802^b^
**Out of Rio de Janeiro**	10 (62.5)	14 (56.0)	
**Rio de Janeiro**	6 (37.5)	11 (44.0)	
**Schooling**			0.7445^b^
**≤ 9 years**	9 (56.3)	15 (60.0)	
**> 9 years**	6 (37.5)	8 (32.0)	
**Unknown**	1 (6.2)	2 (8.0)	

a: Fisher exact test,

b: qui-square test, pv = p-value.

### Treatment and evolution

Table 3 depicts antifungal drugs and dosages used for each patient. Itraconazole was used by all patients at doses ranging from 200 to 600 mg/day. Combination of drugs was required for 78% (n = 32) of the patients. The most widely used therapeutic regimen was intravenous AMB, complemented with oral itraconazole. The median treatment time was 16.7 months (1.5 to 99.2 months). In 73.2% of the cases (n = 30) there was at least one hospitalization, 33.3% (n = 10) motivated by bone sporotrichosis. By the end of the study, 53.7% (n = 22) of patients were considered cured. Among immunocompetent patients, 84.6% (11/13) achieved this outcome, while only 39.3% (11/28) of immunocompromised patients cured. Thus, the calculated risk ratio for an immunosuppressed patient to cure from bone sporotrichosis was 0.35 (i.e., 1/2.84) (95% CI: 0.15–0.82, p = 0.02), compared to an immunocompetent patient (S1 Table). Eight patients (19.5%) were still under treatment, two (4.9%) were lost to follow-up and nine (22.0%) died (mainly related to AIDS).

**Table 3:** Treatment and clinical evolution of patients with bone sporotrichosis treated at the INI-Fiocruz between 1999 and 2016.

**Case**	**Bone involvement**	**Treatment for sporotrichosis**	**Time**^**a**^ **(months)**	Outcome
**1**	Multifocal	ITZ 200mg 12mo, ITZ 400mg 24mo **/** AMB (d): ~ 1g	37.7	Cure (amputation)^b^
**2**	Multifocal	ITZ 200mg 4mo / AMB (d): 9.2, AMB (l): 6.8g / TRB 250mg 1mo / PSZ 8mo	37.6	Death^c^
**3**	Multifocal	ITZ 400mg 12mo, ITZ 200mg 17mo / AMB (d): 6.4g / TRB 250mg 6.5mo	25.9	Cure
**4**	Multifocal	ITZ 200/400mg since August 2011 / AMB (d): 1.5g / TRB 500mg 2mo	68.8	Treating^c^
**5**	Multifocal	ITZ 200mg 1mo / AMB (d): 2.5g, ANF (l): 12.8g / TRB ~ 17mo / PSZ ~15mo	19.4	Death
**6**	Multifocal	ITZ 200mg 2mo, ITZ 400mg 14.5mo / AMB (d): 3.4g, AMB (l) 14.2g / TRB 13mo / PSZ 3mo	44.4	Death^c^
**7**	Multifocal	ITZ 400mg 60mo / ITZ 200mg since September 2017 / AMB (d) 1.5g / TRB 500mg 22mo	48.2	Treating^c^
**8**	Unifocal	ITZ 100mg 3mo, ITZ 200mg 1mo, ITZ 400mg 1.5mo	1.5	Cure (amputation)
**9**	Unifocal	ITZ 400mg 12mo	19.3	Cure
**10**	Unifocal	ITZ 200mg 4mo, ITZ 100mg 3.5mo	3.5	Cure^c^
**11**	Unifocal	ITZ 100mg 1mo, ITZ 200mg 9mo	14.4	Lost to follow-up
**12**	Unifocal	ITZ 200mg 2mo, ITZ 300 mg 14mo / AMB (l): 3.6g	16.7	Cure
**13**	Unifocal	ITZ 400mg 43mo	43	Death
**14**	Unifocal	ITZ 100mg 2mo, ITZ 400mg 6mo	6	Cure
**15**	Unifocal	ITZ 400mg 19mo / AMB (d): 295mg, AMB (l): 1.2g / TRB 250mg 9mo	5.6	Cure (amputation)
**16**	Multifocal	ITZ 400mg 13mo / AMB (d): 500mg, AMB (l): 4.4g / TRB 250mg 4mo	18.2	Cure
**17**	Unifocal	ITZ 200mg 2mo, ITZ 400mg ~7mo	7.1	Cure
**18**	Multifocal	ITZ 100mg ~12mo, ITZ 200/400mg ~5mo / AMB (d): 315mg, AMB (l): 900mg	21.8	Death
**19**	Multifocal	ITZ 400mg 16mo / AMB (d): 500mg	14.5	Cure
**20**	Multifocal	ITZ 200mg 1mo, ITZ 400mg 1mo (abandonment), ITZ 400mg since August 2017 / AMB (d): 3.8g	28.9	Treating^c^
**21**	Unifocal	ITZ 400mg irregular use / AMB (d): 1.85g, AMB (l): ~9.6g / TRB 250mg 1.5mo, TRB 500mg ~3mo / PSZ ~3weeks	8.5	Death^c^
**22**	Multifocal	ITZ 200mg 14mo / AMB (d): 1g	10.5	Cure
**23**	Unifocal	ITZ 400mg 8mo / AMB (d): 2.1g / PSZ since September 2017	15.2	Treating
**24**	Multifocal	ITZ 400mg 14mo / AMB (d): 700mg, AMB (l): 6.9g	14.7	Cure
**25**	Multifocal	ITZ 400/200mg ~12mo (relapse), ITZ 400mg since November 2011 / AMB (d): 2.3g	99.2	Treating
**26**	Multifocal	ITZ 200mg 2mo, ITZ 300mg 10mo / TRB 250/500mg 70mo	93.7	Cure
**27**	Multifocal	ITZ 200mg (irregular), ITZ 400mg 10mo	6.5	Lost to follow-up
**28**	Multifocal	ITZ 600mg 2mo, ITZ 400mg 50mo / AMB (d): ~1g	52.8	Cure
**29**	Multifocal	ITZ 400mg since March 2015 / AMB (d): 400mg, AMB (l): 10.6g / TRB 500mg 16mo	35.8	Treating
**30**	Multifocal	ITZ 400mg 12mo / AMB (d): 2g, AMB (l): 5.5g / TRB 250mg 2mo	12.1	Cure
**31**	Multifocal	ITZ 400mg ~16mo / AMB (d): ~8.3g	26.1	Cure
**32**	Unifocal	ITZ 200mg 2mo / ITZ 400mg 12mo / AMB (d): 320mg, AMB (l): 2.2g	15.3	Cure
**33**	Unifocal	ITZ 200mg 4mo, ITZ 400mg 5mo	5	Cure
**34**	Unifocal	ITZ 400mg 9mo / AMB (d): 150mg, AMB (l): 3.6g	13.9	Death
**35**	Unifocal	ITZ 400mg 5.5mo / AMB (d): 1g	14.2	Cure
**36**	Multifocal	ITZ 200mg 1mo, ITZ 400mg 22mo / AMB (d): 4.1g / TRB 250mg ~ 20mo	24.1	Cure
**37**	Multifocal	ITZ 200mg 3mo / AMB (d): 2g	3.5	Death
**38**	Multifocal	ITZ 400mg since November 2016 / AMB (d): 3.4g, AMB (l): 7.2g	6	Treating^c^
**39**	Unifocal	ITZ 200mg 1.5mo, ITZ 400mg 1mo (abandonment), ITZ 400mg since July 2017	15.4	Treating^c^
**40**	Multifocal	ITZ 200mg 9mo, ITZ 400mg 21mo **/** AMB (d): 300mg, AMB (l): 12.4g	28.3	Death
**41**	Multifocal	ITZ 400mg 21mo / AMB (d): ~1.2g	21.3	Cure

ITZ: itraconazole; AMB: amphotericin B; (d) deoxycholate; (l) lipid formulation; ~: approximate dose; TRB: terbinafine; PSZ: posaconazole; g: grams; mg: milligrams; mo: month(s);

a: total time of treatment for bone sporotrichosis;

b: self-amputation of one phalanx but with multifocal involvement;

c: multiple abandonment or irregular treatment.

Among the 22 cured patients, we had access to the initial ESR and CRP measures of 15 and 13 patients, respectively, and both markers in 12 patients (S2 Table). In general, these markers were elevated at the onset of the condition and tended to decrease (median: -50% [interquartile range (IQR): -73.7%;2.6%] for the ESR and -77.6% [IQR: -89.5%;-49.7%] for the CRP) over the course of treatment.

Sequelae occurred in five (12.2%) patients. Amputations occurred in case 8, already described, case 15, also due to an intense destruction of the fourth right finger, despite of 12 months of itraconazole use (400 mg/day) and case 1, the patient ripped out his own necrotic fourth right toe. In case 26, due to osteoarticular sporotrichosis, the patient had bilateral ankylosis of knees, becoming wheelchair bound, while case 10 lost soft tissues of the second right finger, with impairment of movements, also with ankylosis.

In the multivariate analysis, a higher ratio of cure was associated to unifocal bone involvement, 4.84 (95% CI: 1.79–13.08); p < 0.01, compared to multifocal involvement, and white color, 2.49 (95% CI: 0.99–6.28); p = 0.05, compared to non-white patients.

## Discussion

Sporotrichosis is an expanding zoonotic hyperendemia in the state of Rio de Janeiro, Brazil, mainly affecting women in their forties, who keep contact with sick cats and perform peri domiciliary activities [2,3,25]. *Sporothrix brasiliensis* is associated with atypical and potentially severe cases, probably due to its greater virulence compared to other species of the genus [26–27]. The exclusive molecular identification of *S*. *brasiliensis* corroborates the predominance of this species within this region, and its role in severe clinical cases [26]. This study, including 41 patients with bone sporotrichosis over 18 years, is the largest worldwide institutional series on the subject.

The cases evaluated and reported came mainly from areas with low socioeconomic conditions in the metropolitan region of Rio de Janeiro, following the distribution of the so called sporotrichosis "belt" [25]. Since 2013, sporotrichosis became a compulsory reportable disease in the state of Rio de Janeiro, and the clinical support for the patients with sporotrichosis was decentralized, with only the most serious cases being referred to the INI-Fiocruz. This reflected in the increase of cases with bone involvement in this institution, probably with an important selection bias, represented by the 9% of cases with bone involvement seen in 2016 (Graph 1). It draws our attention, the immunosuppression present in our cohort, not only due to HIV infection, but also to alcoholism and corroborates that immunosuppression is a factor associated with invasive forms and bone involvement in sporotrichosis [2,16]. It is worth noting that sporotrichosis was a key to HIV diagnosis in many patients, and that most of the patients were not on antiretroviral therapy, highlighting the opportunistic behavior of sporotrichosis and the importance of investigating disseminated disease. We believe that a maintained hyperendemia of sporotrichosis leads to the overlap with the HIV pandemic that also reflects in a predominance of men and a lower median age in this group. Other different remarkable aspects are the large percentage of non-white HIV patients with low schooling that can be understood as indicators of vulnerability of the population exposed to both diseases, culminating in more severe cases of sporotrichosis [28,29]. Alcoholism is another recognized risk factor for disseminated disease and bone involvement [16]. Previous reviews also highlighted the presence of comorbidities: Gladstone and Littman [6] found 27.2% of comorbidities among the 22 cases reviewed, with alcoholism present in 4.5% of them. Lederer et al. [13] reported a case of bone sporotrichosis, reviewed other 20 cases from 1980 to 2015 and found 52% of comorbidities, with alcoholism present in 23.8% and HIV infection in 14.2%. In our study, besides HIV infection, it was difficult to assess the impact of alcoholism alone, as the casuistic size is small, and more than half of these alcoholics also had HIV coinfection. Gregory et al. [30] demonstrated the potential deleterious clinical effects of the overlap of these two immunosuppressive conditions. Besides, alcoholism affects the therapeutic adherence, which may directly impair the clinical evolution.

The initial mycological diagnosis was established by culture of exudate or fragment of cutaneous lesion, non-invasive and easy to perform tests. Bone biopsy in the context of osteomyelitis is indicated mainly to confirm the etiological agent and guide the correct treatment, being essential in cases of isolated osteoarticular involvement without cutaneous lesions [9]. In the four cases that this procedure was performed, the diagnosis of cutaneous sporotrichosis was previously known.

Radiography was the most used exam for screening and diagnosis of osteomyelitis. Although less sensible than scintigraphy (important for screening), computed tomography and magnetic resonance (for diagnosis), it is less expensive and available in our institution.

Two distinct clinical presentations were observed: unifocal bone involvement by contiguity of cutaneous lesions, mainly in women without immunosuppression, like the zoonotic profile of sporotrichosis in Rio de Janeiro; and asymptomatic multifocal bone involvement, in immunosuppressed men. The bones of the hands were the most affected ones, probably because cats usually scratch and bite the hands of people taking care of them. The feet had an important differential percentage in multifocal involvement, in relation to the unifocal form, and the tibia lesions were present only in the multifocal form, hence the importance to search bone involvement in these sites in disseminated sporotrichosis. We recommend a special attention to the feet, when searching for bone disease in patients with disseminated sporotrichosis, based on our findings herein presented and on the significant odds ratio encountered. In previous reviews, the tibia was the most affected bone [6,13], present in unifocal and multifocal disease.

The combination of AMB and itraconazole was the most used therapeutic option. Doses and time of treatment were individualized mainly in multifocal forms. In several cases, the cumulative dose of AMB was higher than that usually recommended in the literature, as well as the time of itraconazole use [16]. Terbinafine was used as a therapeutic option mostly in cases of drug interactions or intolerance to itraconazole, while posaconazole was used in isolated, severe cases, in which the central nervous system was simultaneously affected. Clinical cure was associated with the zoonotic profile of white patients and unifocal presentation. Furthermore, the lower cure rate among the patients with the multifocal presentation probably reflects the high percentage of immunosuppression in this group, mainly patients with HIV who do not adhere to the treatment, presenting severe conditions requiring multiple admissions and long follow-up periods. These data show how important strategies for adherence to treatment in selected groups are. Also, the need for new affordable antifungals, with good bone penetration, tolerable adverse effects, and less drug interactions. In the literature, several antifungal regimens for the treatment of osteomyelitis have been reported, with cure or improvement in most of them.

The ESR and CRP trend to decrease suggests a relationship between these markers and bone disease activity, something established for bacterial osteomyelitis [31]. Nevertheless, because this is a retrospective study in which we did not obtain data from the entire sample and many patients presented other infectious comorbidities, such as HIV, this analysis may be compromised. It seems reasonable to recommend the measurement of both markers in the follow-up of patients with bone sporotrichosis, but in cases of divergence between them and images, the later should prevail. A prospective study with many cases, strict follow-up, and measurements may help to answer this question, but this would probably demand a multicenter research.

Those with relapse were associated with involvement of several bones and immunosuppression, as reported in a review [13]. Aesthetic, functional and disabling sequelae occurred in five patients. In the literature, there are few data focusing on sequelae from bone sporotrichosis. Gladstone et al. [6] reported cases with the need for surgical debridement and highlighted a permanent articular dysfunction following osteoarticular involvement.

This study presents limitations inherent to a retrospective study using secondary data, of patients treated over 18 years in a reference hospital. However, the observation of this cohort allowed us to ratify the bone sporotrichosis as a challenging chronic condition, with prolonged course of treatment, often with poor results. So, it is particularly important to early diagnose patients with both presentations, unifocal and multifocal osteomyelitis, and prompt an appropriate treatment, to obtain cure, without sequelae.

## Supporting information

S1 Table Univariate and multivariate analyses of possible predictors to cure, of the patients with bone sporotrichosis treated at the INI-Fiocruz between 1999 and 2016. (DOCX)

S2 Table Erythrocyte sedimentation rate (mm/h) and high sensitivity C-reactive protein (mg/dl) values at the onset of the disease and at the end of treatment, in cured patients with bone sporotrichosis, followed up at INI-Fiocruz, from 1999 to 2016. (DOCX)

S1 STROBE Checklist Strobe checklist. (DOC)
