# Supplementary material for: Bone sporotrichosis: 41 cases from a reference hospital in Rio de Janeiro, Brazil
Source: PLoS Negl Trop Dis. 2021 Mar 17;15(3):e0009250. doi: 10.1371/journal.pntd.0009250 (PMC8007180; doi:10.1371/journal.pntd.0009250)
Supplement: S1 Table — (DOCX) [file pntd.0009250.s001.docx]

Supplementary table 1: Univariate and multivariate analyses of possible predictors to cure, of the patients with bone sporotrichosis treated at the INI-Fiocruz between 1999 and 2016.

|  | Descriptive  N (%) | | Univariate | | Multivariate | |
| --- | --- | --- | --- | --- | --- | --- |
|  | Not cured | Cured | CRR (CI) | pv | CRR (CI) | pv |
| Total | 19 (46.3) | 22 (53.7) |  |  |  |  |
| Immunosuppression |  |  |  |  |  |  |
| Yes | 17 (60.7) | 11 (39.3) | 1 |  |  |  |
| No | 2 (15.4) | 11 (84.6) | 2.84  (1.22-6.59) | **0.01** |  |  |
| Sex |  |  |  |  |  |  |
| Male | 13 (54.2) | 11 (45.8) | 1 |  |  |  |
| Female | 6 (35.3) | 11 (64.7) | 2.45  (1.02-5.9) | **0.04** |  |  |
| Bone involvement |  |  |  |  |  |  |
| Multifocal | 13 (52) | 12 (48) | 1 |  | 1 |  |
| Unifocal | 6 (37.5) | 10 (62.5) | 4.06  (1.58-10.41) | **<0.01** | 4.84  (1.79-13.08) | **<0.01** |
| Skin color |  |  |  |  |  |  |
| Non-white | 16 (53.3) | 14 (46.7) | 1 |  | 1 |  |
| White | 3 (27.3) | 8 (72.7) | 1.97  (0.8-4.82) | **0.14** | 2.49  (0.99-6.28) | **0.05** |
| Cat bite |  |  |  |  |  |  |
| Yes | 2 (50) | 2 (50) | 1 |  |  |  |
| No | 17 (45.9) | 20 (54.1) | 1.37  (0.32-5.91) | 0.68 |  |  |
| HIV infection |  |  |  |  |  |  |
| Yes | 12 (57.1) | 9 (42.9) | 1 |  |  |  |
| No | 7 (35) | 13 (65) | 2.07  (0.88-4.89) | **0.1** |  |  |
| Alcoholism |  |  |  |  |  |  |
| Yes | 4 (44.4) | 5 (55.6) | 1 |  |  |  |
| No | 15 (46.9) | 17 (53.1) | 0.71  (0.25-1.98) | 0.51 |  |  |

CRR: Cure rate ratio; CI: Confidence interval; pv: p-value.
