# Supplementary material for: Bone sporotrichosis: 41 cases from a reference hospital in Rio de Janeiro, Brazil
Source: PLoS Negl Trop Dis. 2021 Mar 17;15(3):e0009250. doi: 10.1371/journal.pntd.0009250 (PMC8007180; doi:10.1371/journal.pntd.0009250)
Supplement: S2 Table — (DOCX) [file pntd.0009250.s002.docx]

Supplementary table 2: Erythrocyte sedimentation rate (mm/h) and high sensitivity C-reactive protein (mg/dl) values at the onset of the disease and at the end of treatment, in cured patients with bone sporotrichosis, followed up at INI-Fiocruz, from 1999 to 2016.

| CASE | Initial ESR | Final ESR | Initial hs-CRP | Final hs-CRP |
| --- | --- | --- | --- | --- |
| 1 | 57 | 25 | 4,59 | 0.8 |
| 3 | 70 | 90 | 1.1 | 0.3 |
| 10 | 70 | 35 | NM | 0.23 |
| 12 | 19 | 20 | 0.3 | 0.1 |
| 15 | 75 | 22 | 4.89 | NM |
| 17 | 23 | 15 | NM | <6^1^ |
| 19 | 140 | 60 | 0.1 | 0.1 |
| 22 | 85 | 120 | 2.8 | 5.3 |
| 24 | 120 | 28 | 16.3 | 0.19 |
| 26 | 56 | 80 | 1.78 | 0.6 |
| 30 | 140 | 70 | 9.35 | 0.7 |
| 32 | 140 | 5 | 0.6 | 0.1 |
| 33 | 19 | 19 | 0.4 | 1.7 |
| 36 | 140 | 15 | 6.1 | 0.7 |
| 41 | 90 | 10 | 14.2 | 1.1 |

ESR: Erythrocyte sedimentation rate (normal if ≤ 10 mm/h for men and ≤ 15 mm/h for women); hs-CRP: high sensitivity C-reactive protein (normal if ≤ 0.3 mg/dl); 1: The only patient for whom the measured CRP was traditional (non-high sensitivity; normal if <6 mg/dl); NM: Not measured.
