# Supplementary material for: Bone sporotrichosis: 41 cases from a reference hospital in Rio de Janeiro, Brazil
Source: PLoS Negl Trop Dis. 2021 Mar 17;15(3):e0009250. doi: 10.1371/journal.pntd.0009250 (PMC8007180; doi:10.1371/journal.pntd.0009250)
Supplement: S1 STROBE Checklist — (DOC) [file pntd.0009250.s003.doc]

STROBE Statement—checklist of items that should be included in reports of observational studies

|  | | Item No | Recommendation |
| --- | --- | --- | --- |
| **Title and abstract** | | 1 | *(*a) Indicate the study’s design with a commonly used term in the title or the abstract  The term is indicated in the abstract, as follows: We studied a retrospective cohort of 41 cases of bone sporotrichosis, diagnosed from 1999-2016. |
|  |  |  | (*b*) Provide in the abstract an informative and balanced summary of what was done and what was found. This is done in the abstract. |
| Introduction | | | |
| Background/rationale | | 2 | Explain the scientific background and rationale for the investigation being reported. Available with relevant references. |
| Objectives | | 3 | State specific objectives, including any prespecified hypotheses.  Lines 73-75: We evaluated the socio-demographic and epidemiological characteristics, and the clinical evolution of the patients with bone sporotrichosis, in a reference hospital, aiming to describe the cases and to find explanatory variables. |
| Methods | | | |
| Study design | | 4 | Present key elements of study design early in the paper  Please, see section “Place of study, patients, and study design”, lines 77-83. |
| Setting | | 5 | Describe the setting, locations, and relevant dates, including periods of recruitment, exposure, follow-up, and data collection  Please, see section “Place of study, patients, and study design”, lines 77-83. |
| Participants | | 6 | (*a*) *Cohort study*—Give the eligibility criteria, and the sources and methods of selection of participants. Describe methods of follow-up  Please, see section “Place of study, patients, and study design”, lines 77-83.  *Case-control study*—Give the eligibility criteria, and the sources and methods of case ascertainment and control selection. Give the rationale for the choice of cases and controls  *Cross-sectional study*—Give the eligibility criteria, and the sources and methods of selection of participants |
|  |  |  | (*b*) *Cohort study*—For matched studies, give matching criteria and number of exposed and unexposed. Not applicable.  *Case-control study*—For matched studies, give matching criteria and the number of controls per case |
| Variables | | 7 | Clearly define all outcomes, exposures, predictors, potential confounders, and effect modifiers. Give diagnostic criteria, if applicable  These data are presented from lines 91 through 122, within sections “**Patient management”, “Molecular identification”, “Definition of bone sporotrichosis” and “Data collection and statistical analysis”.** |
| Data sources/ measurement | | 8* | For each variable of interest, give sources of data and details of methods of assessment (measurement). Describe comparability of assessment methods if there is more than one group  Available in lines 111-122, within the section **“Data collection and statistical analysis”.** |
| Bias | | 9 | Describe any efforts to address potential sources of bias  Partially addressed in the “Data collection and statistical analysis” and later in the “Discussion” section. |
| Study size | | 10 | Explain how the study size was arrived at  This was an institutional cohort, with its size obtained after the review of cases who fulfilled an inclusion criterion, a convenience sample. This explanation is given in lines 77-83. |
| Quantitative variables | | 11 | Explain how quantitative variables were handled in the analyses. If applicable, describe which groupings were chosen and why  Available in lines 111-122, within the section **“Data collection and statistical analysis”.** |
| Statistical methods | | 12 | (*a*) Describe all statistical methods, including those used to control for confounding  Available in lines 111-122, within the section **“Data collection and statistical analysis”.** |
|  |  |  | (*b*) Describe any methods used to examine subgroups and interactions  Available in lines 111-122, within the section **“Data collection and statistical analysis”.** |
|  |  |  | (*c*) Explain how missing data were addressed  Available in lines 111-122, within the section **“Data collection and statistical analysis”.** |
|  |  |  | (*d*) *Cohort study*—If applicable, explain how loss to follow-up was addressed  Available in lines 111-122, within the section **“Data collection and statistical analysis”.**  *Case-control study*—If applicable, explain how matching of cases and controls was addressed  *Cross-sectional study*—If applicable, describe analytical methods taking account of sampling strategy |
|  |  |  | (*e*) Describe any sensitivity analyses Not applicable. |
| Results | | | |
| Participants | 13* | (a) Report numbers of individuals at each stage of study—eg numbers potentially eligible, examined for eligibility, confirmed eligible, included in the study, completing follow-up, and analysed  Lines 125-127. | |
|  |  | (b) Give reasons for non-participation at each stage  Some analyses were performed with a limited number of participants, detailed in lines 164-182. | |
|  |  | (c) Consider use of a flow diagram  We opted not to use a diagram, due to the simple numbers. | |
| Descriptive data | 14* | (a) Give characteristics of study participants (eg demographic, clinical, social) and information on exposures and potential confounders  Available in lines 133-152. | |
|  |  | (b) Indicate number of participants with missing data for each variable of interest  Indicated in lines 164-182, as well as within the tables. | |
|  |  | (c) *Cohort study*—Summarise follow-up time (eg, average and total amount)  Found in lines 251-271 and table 3. | |
| Outcome data | 15* | *Cohort study*—Report numbers of outcome events or summary measures over time  Found in lines 251-271 and table 3. | |
|  |  | *Case-control study—*Report numbers in each exposure category, or summary measures of exposure Not applicable. | |
|  |  | *Cross-sectional study—*Report numbers of outcome events or summary measures Not applicable. | |
| Main results | 16 | (*a*) Give unadjusted estimates and, if applicable, confounder-adjusted estimates and their precision (eg, 95% confidence interval). Make clear which confounders were adjusted for and why they were included  In lines 213-215, 216-219, 259-262, and table 2. | |
|  |  | (*b*) Report category boundaries when continuous variables were categorized  Lines 136-138 and table 3. | |
|  |  | (*c*) If relevant, consider translating estimates of relative risk into absolute risk for a meaningful time period. Not applicable. | |
| Other analyses | 17 | Report other analyses done—eg analyses of subgroups and interactions, and sensitivity analyses.  Multivariate analyses are reported in lines 269-271. | |
| Discussion | | | |
| Key results | 18 | Summarise key results with reference to study objectives.  Theses points are addressed in distinct lines in the discussion. | |
| Limitations | 19 | Discuss limitations of the study, taking into account sources of potential bias or imprecision. Discuss both direction and magnitude of any potential bias  Lines 357-362. | |
| Interpretation | 20 | Give a cautious overall interpretation of results considering objectives, limitations, multiplicity of analyses, results from similar studies, and other relevant evidence  Throughout the discussion and in lines 357-362. | |
| Generalisability | 21 | Discuss the generalisability (external validity) of the study results  Lines 357-358. | |
| Other information | | | |
| Funding | 22 | Give the source of funding and the role of the funders for the present study and, if applicable, for the original study on which the present article is based  Lines 364-369. | |

*Give information separately for cases and controls in case-control studies and, if applicable, for exposed and unexposed groups in cohort and cross-sectional studies.

**Note:** An Explanation and Elaboration article discusses each checklist item and gives methodological background and published examples of transparent reporting. The STROBE checklist is best used in conjunction with this article (freely available on the Web sites of PLoS Medicine at http://www.plosmedicine.org/, Annals of Internal Medicine at http://www.annals.org/, and Epidemiology at http://www.epidem.com/). Information on the STROBE Initiative is available at www.strobe-statement.org.
